# Seasonality of Influenza A(H7N9) Virus in China—Fitting Simple Epidemic Models to Human Cases

**DOI:** 10.1371/journal.pone.0151333

**Published:** 2016-03-10

**Authors:** Qianying Lin, Zhigui Lin, Alice P. Y. Chiu, Daihai He

**Affiliations:** 1 Department of Applied Mathematics, Hong Kong Polytechnic University, Hong Kong (SAR) China; 2 School of Mathematical Science, Yangzhou University, Yangzhou, 225002, People Republic of China; University of Waterloo, CANADA

## Abstract

**Background:**

Three epidemic waves of influenza A(H7N9) (hereafter ‘H7N9’) human cases have occurred between March 2013 and July 2015 in China. However, the underlying transmission mechanism remains unclear. Our main objective is to use mathematical models to study how seasonality, secular changes and environmental transmission play a role in the spread of H7N9 in China.

**Methods:**

Data on human cases and chicken cases of H7N9 infection were downloaded from the EMPRES-i Global Animal Disease Information System. We modelled on chicken-to-chicken transmission, assuming a constant ratio of 10^−6^ human case per chicken case, and compared the model fit with the observed human cases. We developed three different modified Susceptible-Exposed-Infectious-Recovered-Susceptible models: (i) a non-periodic transmission rate model with an environmental class, (ii) a non-periodic transmission rate model without an environmental class, and (iii) a periodic transmission rate model with an environmental class. We then estimated the key epidemiological parameters and compared the model fit using Akaike Information Criterion and Bayesian Information Criterion.

**Results:**

Our results showed that a non-periodic transmission rate model with an environmental class provided the best model fit to the observed human cases in China during the study period. The estimated parameter values were within biologically plausible ranges.

**Conclusions:**

This study highlighted the importance of considering secular changes and environmental transmission in the modelling of human H7N9 cases. Secular changes were most likely due to control measures such as Live Poultry Markets closures that were implemented during the initial phase of the outbreaks in China. Our results suggested that environmental transmission via viral shedding of infected chickens had contributed to the spread of H7N9 human cases in China.

## Introduction

The first human case of influenza A(H7N9) infection (hereafter ‘H7N9’) was identified in eastern China on 31 March 2013. As of 17 July 2015, there were a total of 677 laboratory confirmed human cases of infection and at least 275 reported deaths [1]. H7N9 had spread to other parts of mainland China, Hong Kong, Taiwan and Canada. These human cases were characterized by three major epidemic waves: Wave I from early to mid 2013, Wave II from late 2013 to mid 2014, and Wave III from late 2014 to early 2015. It is of great public health importance to reveal the seasonality in these human epidemic waves.

H7N9 establishment and transmission among birds and transmission among mammals had been well-studied, but scientific evidence on human-to-human transmission was conflicting. H7N9 was originated from Baer’s Pochard, a duck species, in Hunan Province in 2010 and duck influenza viruses in Nanchang city in 2000 [2]. Lam et al. suggested that probably due to poultry movement and trade that the H7N9 influenza virus showed genetic diversity, became established in chickens and then disseminated into wider regions [3]. Experimental studies on mice, ferrets and pigs confirmed the transmissibility through direct contact and limited airborne spreading via mammals [4–9]. A number of studies investigated into human-to-human transmission. Peng et al. used an evolutionary dynamic model to predict that the H7N9 virus would acquire properties for human-to-human transmission in 11.3 years [2]. Xu et al. studied the structural basis of H7N9 virus and concluded that it was poorly adapted for human-to-human transmission [10]. However, Shi et al. used epidemiological and clinical data from six clustered patients in Shanghai to show that two patients were involved in familial aggregation [11]. A more recent epidemiological investigation also showed that human-to-human transmission between two patients might have occurred in a hospital setting [12].

Previous studies employed mathematical modelling techniques to explore the transmission and geographical distribution of H7N9 infections. Xiao et al. fitted a mathematical model to human cases and suggested that human-to-human transmission potential was limited [13]. Hsieh et al. used a compartmental modelling framework to study both bird-to-bird and bird-to-human transmission and suggested a low level of transmission of the latter [14]. Zhu and Peterson characterized the environmental drivers of H7N9 virus transmission using the Normalized Difference Vegetation Index (NDVI), and concluded that central-Eastern China was a high risk area for the spread of H7N9 [15]. Gilbert et al. created a geographical dataset of live poultry markets and their environmental correlates in China, and by using a statistical model, they accurately predicted the risks of H7N9 human infection in China [16].

Environmental transmission is an important indirect transmission route to be considered. Rohani et al. used a stochastic model to demonstrate how ignoring the environmental transmission route of low pathogenicity avian influenza viruses(LPAIs) could lead to under-estimation of both duration and magnitude of the epidemics [17]. Other studies also demonstrated the presence of avian influenza viruses in the environment [18–22]. Wang et al. collected environmental samples in Zhejiang province, and demonstrated the presence of the H7N9 virus, which oscillated seasonally with peaks in spring and winter. Brown et al. showed that under experimental conditions, factors such as temperature, pH and salinity played a role in the persistence of Avian influenza viruses in water [23, 24]. Viruses persisted in low temperature (i.e. below 17°C), slightly alkaline pH (i.e. ranges 7.4–8.2) and fresh water (ranges 0–20,000 parts/million ppm) conditions. A flexible range for persistent days in water of LPAIs was shown, and the optimal temperature for persistence was 4°C with more than 200 days. Correspondingly, the temperature of Eastern China in spring 2013, winter 2014 and 2015 winter ranges were from −4°C to 31°C (http://www.wunderground.com/).

Previous studies focus on the first two waves of H7N9 human cases in 2013 and 2014. This study presented the results of our mathematical modelling which considered all three epidemic waves of H7N9 which occurred between March 2013 and July 2015 in China. We used compartmental models to investigate whether the spread of H7N9 is affected by seasonality, secular changes and environmental transmission. We applied the plug-and-play inference methods to perform model fit of all three waves of H7N9 cases among humans.

## Materials and Methods

### 0.1 Data

Data for avian influenza H7N9 human cases and chicken cases were downloaded from EMPRES-i Global Animal Disease Information System (http://empres-i.fao.org; accessed on July 10, 2015).

**Fig 1:**
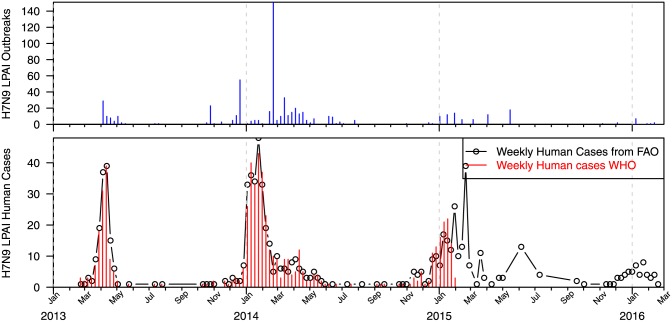
Weekly total of H7N9 outbreaks and laboratory confirmed H7N9 infected human cases.

Fig 1 shows the weekly total of outbreak events (mainly among chicken) related to H7N9 avian influenza and a comparison of weekly total of human cases with H7N9 from two sources. Since the H7N9 is of low pathogenicity, the outbreak events among chicken are most likely under-reported. The number of human cases had been consistent and had shown three waves so far. In this study, we focused on the human cases.

### 0.2 Model Description

We modified the Susceptible-Exposed-Infected-Recovered-Susceptible (SEIRS) model by adding an environmental class, *V*. The model can be described by the following equations:

$$\begin{array}{ccc} \dot{S} & = & \lambda R-\beta S(I+V), \end{array}$$ (1)

$$\begin{array}{ccc} \dot{E} & = & \beta S(I+V)-\sigma E, \end{array}$$ (2)

$$\begin{array}{ccc} \dot{I} & = & \sigma E-\gamma I, \end{array}$$ (3)

$$\begin{array}{ccc} \dot{V} & = & \eta I-\kappa V, \end{array}$$ (4)

$$\begin{array}{ccc} \dot{R} & = & \gamma I-\lambda R, \end{array}$$ (5)

where *β* is the transmission rate, *σ* is the rate at which exposed chickens are infected, and *γ* is the rate at which infected chickens recovered.

The weekly number of cases are:

$$\begin{array}{c} Z_{t}=\int_{\text{a}\;\text{week}}\rho \gamma Idt, \end{array}$$ (6)

where *ρ* is the actual chicken case to reported human case ratio. Without the *V* class, the reduced model is a classical SEIRS model.

Eq (4) was referenced from Rohani et al., confirming the effect of environmental transmission in low pathogenicity avian influenza viruses by simulating the levels of viruses from a lake [17]. Here, we consider *V* as a shadow class, in which infected farm chickens leave their infectious copies to mimic the fact that infected farm chickens cast viruses into the environment. We assume that as the number of infected chickens go up, the amount of viruses being shed into the environment will also go up [17]. These viruses will likely spread among wild birds and will likely persist for a certain period in the environment. These infectious copies were imported at a rate of *η* and cleared up at a rate of *κ*. By this consideration, class *V* is able to synthesize all environmental transmission modes.

Eqs (1) to (5) are stated in absolute numbers and we also make a simplifying assumption of a constant population over the two-year period.

Eq (6) was referenced from [25] and we assume the measurement noise follows an over-dispersed Poisson distribution. In particular, we assume the confirmations follow a Poisson process. In addition, the rate of the Poisson process is a Gamma random variable. Thus the observed weekly laboratory-based confirmations *C*_*t*_ is a random sample from a Negative-binomial (NB) distribution

$$\begin{array}{c} C_{t}∼\text{NB}\left(\text{size}=\frac{1}{\tau },\text{prob.}=\frac{1}{1+Z_{t}\tau }\right) \end{array}$$ (7)

where *τ* is an over-dispersion parameter which will be estimated. We denote the likelihood function for the week *t* as *l*_*t*_ which is the ‘probability’ (density) of observing *C*_*t*_, given *Z*_*t*_ and *τ*, under the NB distribution [25]. The overall likelihood for the whole time series is [26]

$$\begin{array}{c} L\left(\theta |C_{0,...,N}\right)=\prod_{t=0}^{N}\;l_{t}, \end{array}$$ (8)

where *θ* is denoted as the parameter vector. We use the iterated filtering method within the plug-and-play likelihood inference framework to estimate the maximum likelihood estimates for *θ*. This method has been extensively studied and used in a number of publications [27–33]. We apply the plug-and-play likelihood-based inference framework [32] in the procedure summarized as follows. First, we simulate the model (Eqs (1–6)) using the fixed time-step Euler-multinomial algorithm, which will capture the demographic noise. Second, we take into account the measuremental noise via Eqs (6–7). Third, we compute the likelihood estimation for our model given the observed data using Sequential Monte Carlo (SMC) technique and then maximize them using the Iterated Filtering Method [27]. Fourth, we compare different models using the second-order for both small-sample-size corrected Akaike’s Information Criterion (*AICc*) and Bayesian Information (*BIC*) [34, 35].

The *AICc*[31] was used to measure the goodness-of-fit of models:

$$\begin{array}{c} AICc=-2\log L+2k+\frac{2k(k+1)}{N-k-1}, \end{array}$$ (9)

where *N* is denoted as the number of data points and *k* is denoted as the number of free parameters.

### Model Fitting

We made the following assumptions about the model input parameters: We assumed the ratio of human case to avian case to be fixed at 10^−6^: 1 [14]. We fixed the size of the farm chicken populations to be 10^9^, the mean latent period (*σ*^−1^) to be 2 days, and the mean infectious period (*γ*^−1^) to be 6 days. We assumed the replenishment rate (*λ*) to be 0.5 year, 1.5 year and 2.5 years which is the mean lifetime of farm chickens. This rate referred to removal due to slaughtering for sales, death and loss of immunity. We further assumed the transmission rate is a flexible function, i.e., a cubic spline function with *n*_*β*_ nodes uniformly distributed either in a year (periodic) or over the whole time interval of the time series (non-periodic). We fitted the model with the number of nodes to be 7, 8, 9, ⋯ , 14. We fitted three types of models: a non-periodic model with an environmental class, a non-periodic model without an environmental class, and a periodic model with an environmental class. For each type of model, we fitted 24 different combinations of number of nodes (7 to 14) and values of mean lifetime of farm chicken (0.5, 1.5 and 2.5 years).

Statistical inference for partially-observed Markov processes—POMP (http://kingaa.github.io/pomp/) was used to fit these models to the weekly laboratory confirmed H7N9 human cases.

## Results

**Fig 2:**
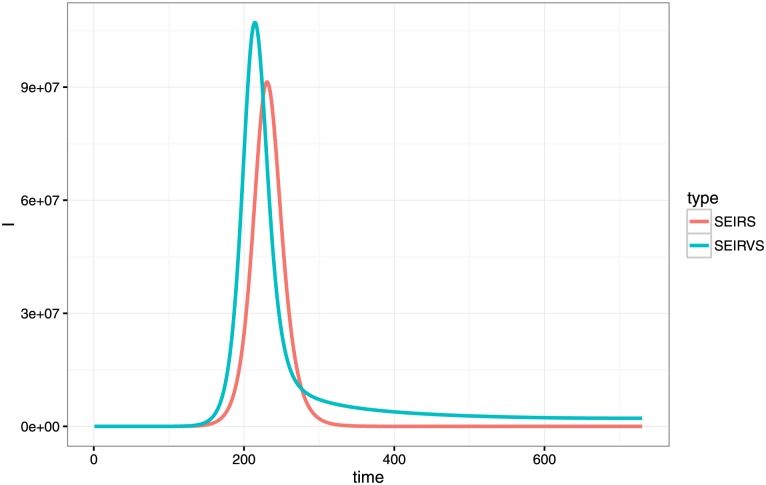
Simulated infection cases using SEIRVS and SEIRS models.

Fig 2 presents the infection simulation results from SEIRVS model and SEIRS model. In this simulation, all parameters of both models are the same and are fixed. Time period is from day 1 to day 830, i.e. 2 years. This figure showed an obvious flat tail of the number of infections in SEIRVS model after the peak, which was also observed in Fig 1 for the second and third waves.

**Fig 3:**
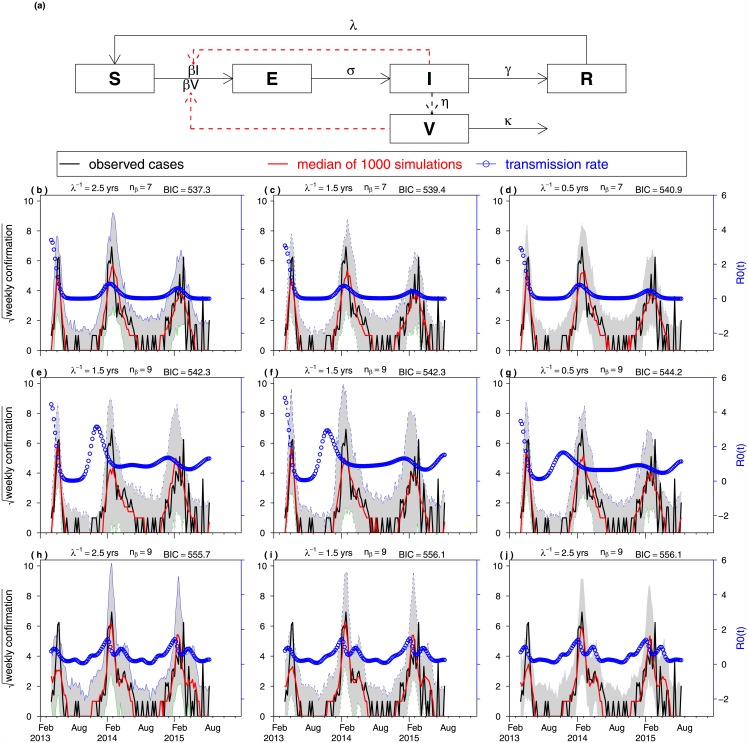
Fitting an SEIRVS model to the weekly human cases of avian H7N9 in China. (a) Model Structure. (b-g) Fitting results with different parameter settings. We computed AICc for a range of *λ* at 0.5, 1.5 and 2.5 years respectively and the range of nodes from 7 to 14. We display the results of the three models with the smallest AICc. (b-d) non-periodic model with an environmental class, (e-g) non-periodic model without an environmental class, and (h-j) periodic model with an environmental class. The model fit became worse in (e-j), as reflected in the *AICc*. Larger *AICc* implies poorer fit.

Fig 3 panel (a) shows the flow diagram of the epidemic model used in the fitting. Panels (b-g) showed the comparisons between observed H7N9 cases (black curve) and model simulations (shaded region and the red curve as the median of 1000 simulations). The curve with blue circles shows the reconstructed transmission rate, in the unit of the basic reproductive number, denoted as R(t). We listed the top three combinations which attained the lowest *AICc* for each model: a non-periodic model with an environmental class (Fig 3 panels b-d), a non-periodic model without an environmental class (panels e-g), and a periodic model with an environmental class (panels h-j). We also listed the top three combinations which attained the lowest *BIC* for each model in Figure A in S1 Appendix.

Tables A and B in S1 Appendix show the estimates of *η* and *κ*. We noted that the viral shedding rate is small and the duration is fairly long. These estimates are also dependent on the choice of *AICc* or *BIC*, by comparing Tables A and B in S1 Appendix. The number of nodes (*n*_*β*_) in the best fitting is smaller when we use *BIC* rather than *AICc*.

## Discussion

In this study, we fitted different compartmental models and we have used the likelihood-based inference technique to model the three epidemic waves of H7N9 human cases that occurred in China from March 2013 to July 2015. We have considered seasonality, secular changes and environmental transmission in these models.

Our results indicated that the model choice with non-periodic (i.e. secular) model for both direct transmission and environmental transmission provided the best model fits for the observed weekly laboratory confirmed human cases in the three epidemic waves. Our best-fitting models had transmission rates that were within biologically plausible ranges.

When our best-fitting model of a periodic transmission model without an environmental class were compared to that of the non-periodic model with an environmental class, there was a model improvement of 6.6 in Δ*AICc*. When we go from a periodic model with an environmental class to a non-periodic model with an environmental class, an improvement of 15.8 in Δ*AICc* was found. Our results suggested the importance of considering environmental transmission and secular changes in the transmission rate. Our best-fitting model also provided estimates for virus-importing rate (*η*) of 1.8 per year and virus-persistence duration (*κ*^−1^) of 115.85 days, both of which were within biologically plausible ranges [23]. We noted that in Tables A and B in S1 Appendix, our non-periodic models with an environmental class (i.e. Models 1–3) are significantly better than the other types of models (i.e. Models 4–9), regardless of the choice of *AICc* and *BIC*. Therefore, our qualitative conclusions still hold when *BIC* is used in model selection.

Our model also provided insights into the cause of these H7N9 waves. Strong public health control measures were implemented in 2013 when the first human H7N9 case was identified. H7N9 virus evolution could have also changed the transmission rate of the virus.

Previous mathematical modelling studies on H7N9 accounted for poultry-to-human transmission [13, 14, 36]. However, none of them use an environmental class in a compartmental model to account for their patterns of spreading. Chowell et al. combined Bayesian approach with a SEIR model to fit to observed data on H7N9. They estimated that the reproduction number was approximately 0.1 [36]. Considering both direct poultry-to-human transmission and human-to-human transmission, Xiao et al estimated the reproduction number for human-to-human transmission to be 0.467 [13] and Hsieh et al. estimated the one for infection among birds to be 4.10 [14]. Consistent with their results, we assumed that human-to-human transmission is limited, which allowed us to draw a simplifying assumption that human cases were at constant proportion to chicken cases across the time period.

There still exist some gaps in the fitting simulation and the observed cases in all of the three waves. We may explain the gap in the peak of second and third waves by proposing that, both of these peaks occurred around the Chinese New Year, when Chinese people are likely to consume more chickens for celebration [37]. However, this theory could not explain the gap in the first wave. There are other limitations in our model. We have adopted simplifying assumptions about virus shedding into the environment. In reality, these viruses could persist and cause outbreaks among wild birds.

**Fig 4:**
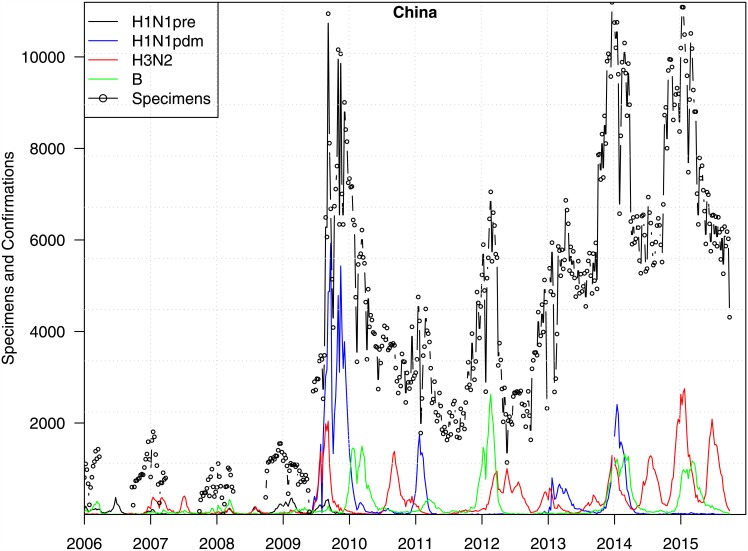
Weekly total of specimens tested for influenza and laboratory confirmed H1N1, H3N2 and influenza B cases.

Future work could explore into the environmental transmission routes of H7N9 in China. By considering the frequent transportation of chickens from the farms to the live poultry markets, a spatio-temporal SEIRVS-Secular model could be established to estimate the rates of change in the Susceptible and Infected compartments of the model. Moreover, further research may include studying the gap between fitting the simulated cases to the observed cases in the first wave. We also look into the changes in testing efforts in China due to the outbreaks of H7N9. Fig 4 shows the total number of specimens processed for influenza and weekly laboratory confirmations of human influenza cases from China from Jan 2006 to May 2015. These testing efforts, as reflected in the total specimens processed for influenza, had been increased significantly twice, i.e. after the 2009 influenza A(H1N1) pandemic and then again after 2013, when the first H7N9 case was identified. Total specimens processed for influenza and weekly laboratory confirmations of human influenza cases from China from Jan 2006 to May 2015 were downloaded from FluNet of the World Health Organization (http://www.who.int/influenza/gisrs_laboratory/flunet/en/; accessed on July 10, 2015).

We concluded that secular changes and environmental transmission have both contributed to the spread in the three epidemic waves of H7N9 in China. The above conclusions are robust against the choice of our model input parameters, such as the number of nodes in the transmission rate and the assumed mean lifetime of farm chickens, both of which are within biologically plausible ranges. Our study has important public health implications: targeted surveillance should consider the secular changes observed in the human cases of H7N9, and environmental routes of transmission should be closely monitored.

## Supporting Information

S1 Appendix **Table A in S1 Appendix**, List of parameter estimates. Models are ordered according to *AICc*. **Table B in S1 Appendix**, List of parameter estimates. Models are ordered according to *BIC*. **Figure A in S1 Appendix**, Fitting an SEIRVS model to the weekly human cases of avian H7N9 in China. (a) Model Structure. (b-g) fitting results with different parameter settings. We computed BIC for a range of *λ* at 0.5, 1.5 and 2.5 years respectively and the range of nodes from 7 to 14. We display the results of the three models with the smallest BIC. (b-d) non-periodic transmission rate with an environmental class, (e-g) non-periodic transmission rate without an environmental class, and (h-j) periodic transmission rate with an environmental class. The model fitting became worse in (e-j), as reflected in the *BIC*. Larger *BIC* implies poorer fitting.
(PDF)
