# Supplementary material for: Seasonality of Influenza A(H7N9) Virus in China—Fitting Simple Epidemic Models to Human Cases
Source: PLoS One. 2016 Mar 10;11(3):e0151333. doi: 10.1371/journal.pone.0151333 (PMC4786326; doi:10.1371/journal.pone.0151333)
Supplement: S1 Appendix — Table A in S1 Appendix, List of parameter estimates. Models are ordered according to AICc. Table B in S1 Appendix, List of parameter estimates. Models are ordered according to BIC. Figure A in S1 Appendix, Fitting an SEIRVS model to the weekly human cases of avian H7N9 in China. (a) Model Structure. (b-g) fitting results with different parameter settings. We computed BIC for a range of λ at 0.5, 1.5 and 2.5 years respectively and the range of nodes from 7 to 14. We display the results of the three models with the smallest BIC. (b-d) non-periodic transmission rate with an environmental class, (e-g) non-periodic transmission rate without an environmental class, and (h-j) periodic transmission rate with an environmental class. The model fitting became worse in (e-j), as reflected in the BIC. Larger BIC implies poorer fitting. (PDF) [file pone.0151333.s001.pdf]

Seasonality of influenza A(H7N9) virus in China – fitting simple epidemic models to human cases

Qianying Lin[1], Zhigui Lin[1,2], Alice P.Y. Chiu[1,*], Daihai He[1]

1 Department of Applied Mathematics, Hong Kong Polytechnic University, Hong Kong (SAR) China.
2 School of Mathematical Science, Yangzhou University, Yangzhou, 225002, People Republic of China.
* alice.py.chiu@polyu.edu.hk

Supplementary Materials

Table S1: *

Table A. List of parameter estimates. Models are ordered according to *AICc*.

model	λ^{-1} (yrs)	η (yrs^{-1})	κ^{-1} (yrs)	n_β	τ	$S(0)$	$I(0)$	$R(0)$	L	AIC_c
1	2.50	1.80	0.43	13	0.12	0.640075	0.000003	0.359915	-235.9	508.8
2	0.50	1.66	0.43	13	0.14	0.625142	0.000004	0.374847	-236.2	509.5
3	1.50	1.66	0.62	13	0.14	0.653989	0.000003	0.346002	-236.4	509.8
4	1.50	1.82	0.50	13	0.24	0.513830	0.000059	0.485993	-241.6	515.0
5	1.50	3.88	0.36	9	0.19	0.525726	0.000066	0.474077	-247.0	515.9
6	1.50	3.88	0.36	9	0.19	0.525726	0.000066	0.474077	-247.0	515.9
7	1.50	0.75	0.72	10	0.28	0.943010	0.001902	0.051284	-247.6	524.5
8	2.50	0.98	1.00	9	0.32	0.930563	0.002282	0.062592	-248.9	524.6
9	2.50	0.83	0.90	10	0.28	0.909312	0.001610	0.085858	-247.7	524.6

(Fixed parameters $N = 10^9$, $\rho = 10^{-6}$, $\sigma = 2$ days, and $\gamma = 6$ days. $E(0) = V(0) = I(0)$.)

Table S2: *

Table B. List of parameter estimates. Models are ordered according to BIC.

model	λ^{-1} (yrs)	η (yrs^{-1})	κ^{-1} (yrs)	n_β	τ	$S(0)$	$I(0)$	$R(0)$	L	BIC
1	2.50	4.94	0.81	7	0.25	0.516642	0.000029	0.483272	-244.5	537.3
2	1.50	3.87	1.01	7	0.27	0.518871	0.000042	0.481003	-245.6	539.4
3	0.50	4.03	0.93	7	0.28	0.876324	0.000054	0.123513	-246.3	540.9
4	1.50	3.88	0.36	9	0.19	0.525726	0.000066	0.474077	-247.0	542.3
5	1.50	3.88	0.36	9	0.19	0.525726	0.000066	0.474077	-247.0	542.3
6	0.50	3.14	0.29	9	0.17	0.934268	0.000032	0.065637	-248.0	544.2
7	2.50	0.98	1.00	9	0.32	0.930563	0.002282	0.062592	-248.9	555.7
8	1.50	0.92	0.67	9	0.31	0.934024	0.002293	0.059098	-249.1	556.1
9	2.50	1.07	0.83	9	0.32	0.911151	0.002104	0.082537	-249.1	556.1

(Fixed parameters $N = 10^9$, $\rho = 10^{-6}$, $\sigma = 2$ days, and $\gamma = 6$ days. $E(0) = V(0) = I(0)$.)

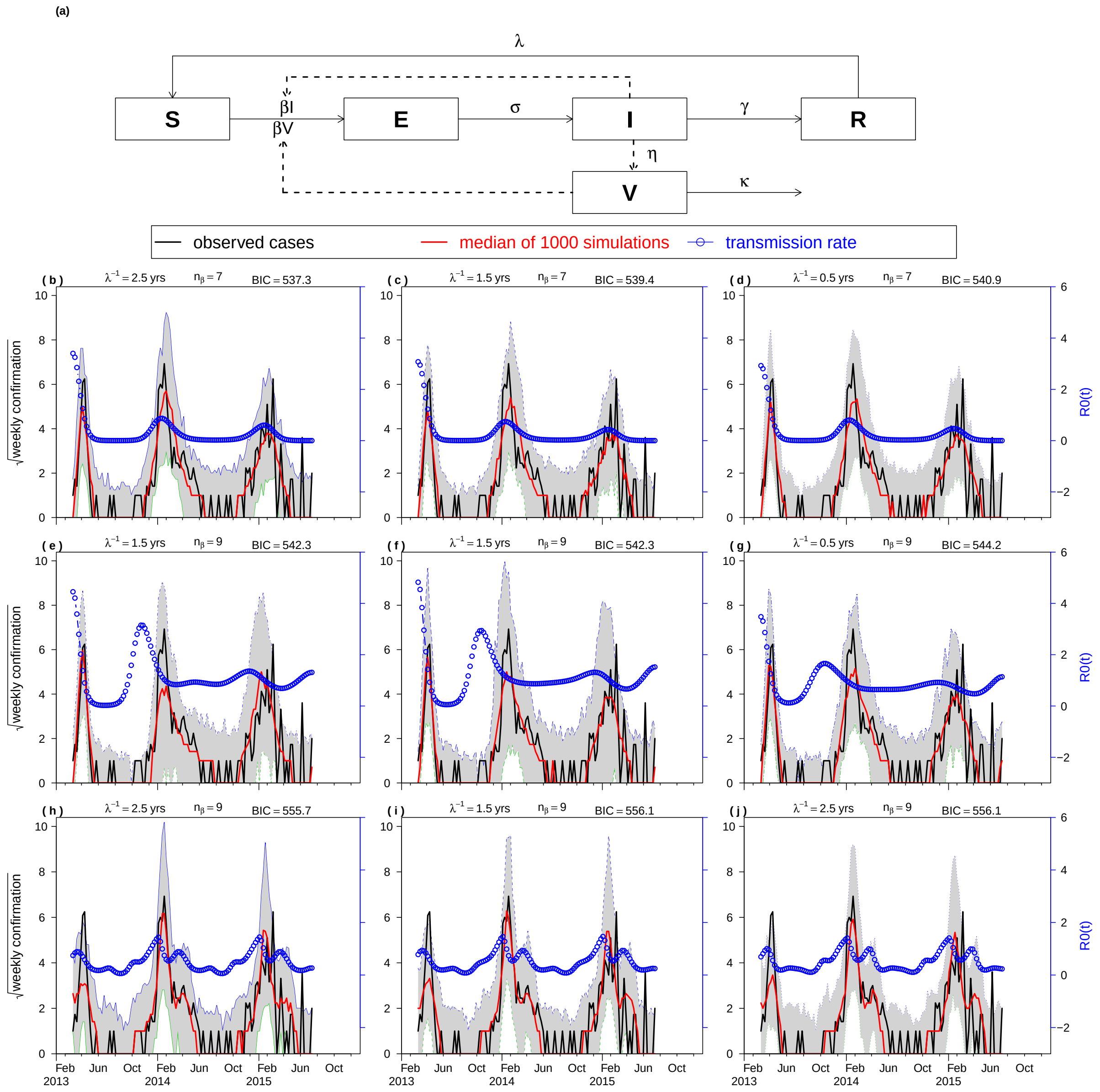

Figure S3: *

Figure A Fitting an SEIRVS model to the weekly human cases of avian H7N9 in China. (a) Model Structure. (b-g) fitting results with different parameter settings. (b-d) non-periodic transmission rate with an environmental class, (e-g) non-periodic transmission rate without an environmental class, and (h-j) periodic transmission rate with an environmental class. The model fitting became worse in (e-j), as reflected in the *BIC*. Larger *BIC* implies poorer fitting.
